# Grunt

**Published:** 1866-12

**Authors:** 


### GRUNT.

Nature, so beautifully appropriate and economical in all her arrangements, makes a double use of the expression of pain or suffering both in men and animals,—a sigh, a moan, a tear, always attract the attention and excite the sympathies of the well and also afford a grateful relief to the sufferer. Those griefs craze the brain which do not vent themselves in weeping. The tearless eye under trouble, breaks the heart. The parent who whips or scolds his child because he cries under suffering or trouble, is a brute or an ignoramus. Neither mirth nor mourning ought to be restrained of their natural expression. Laughter increases the gladness and sighs relieve the sorrowing heart. A very striking exhibition of this idea has frequently come under the observation of the most careless. There is a class of men and women of feeble intellect though they be, who are always complaining; they can entertain you by the hour with the details of their bodily sufferings; according to their own story, they are never free from some ache, or pain, or malady, and you wonder after listening to their interminable narrations that they had not long ago died from the effects of such sufferings; and yet they do not die; they live on, and on, and on, to vex successive generations with their dismal histories; if you sit down to the table with them, you will find that they can eat as much and as fast as the most robust, and have very nice perceptions of the good things of this life, eatable and drinkable. We may, then, reasonably infer that habitual grunting is a life-preserver, and the Frenchman was not very far wrong after all, who advocated the theory several years ago that while it was indisputable that laughing made the body fat, and that good natured children, the lively, merry ones, were healthy, that it was equally certain that crying children were not less so; in other words, that crying did a child good, it promoted a more vigorous circulation of the blood, and helped to develop the lungs.

